# Selective enrollment in Disease Management Programs for coronary heart disease in Germany – An analysis based on cross-sectional survey and administrative claims data

**DOI:** 10.1186/s12913-017-2162-y

**Published:** 2017-04-04

**Authors:** Julia Röttger, Miriam Blümel, Reinhard Busse

**Affiliations:** Department of Health Care Management, Technical University Berlin, and Centre for Health Economics Research, Strasse des 17. Juni 135, 10623 Berlin, Germany

**Keywords:** Disease management program, Chronic care, Chronically ill, Coronary heart disease, Structured care, Germany, Survey data, Administrative claims data, Selective enrollment

## Abstract

**Background:**

In 2002, Disease Management Programs (DMPs) were introduced within the German healthcare system with the aim to increase the quality of chronic disease care. Due to the enrollment procedures, it can be assumed a) that only certain patients actively decide to enroll in a DMP and/or b) that only certain patients get the recommendation for DMP enrollment from their physician. How strong this assumed effect of self- and/or professional selection is, is still unclear.

**Methods:**

We used data from a cross-sectional postal-survey linked on individual level with administrative claims data from a German sickness fund. The sample consisted of individuals suffering from coronary heart disease (CHD) who i) were either enrolled in the respective DMP or ii) fulfilled the disease related criteria for enrollment but were not enrolled. We applied multivariate logistic regression analyses to assess factors on patient level associated with DMP enrollment.

**Results:**

We included 7070 individuals in our analyses. Male sex, higher age and receiving old age pension, a higher Charlson Score and a diagnosis of type 2 diabetes increased the odds for DMP-CHD enrollment significantly. Individuals with a diagnosed myocardial infarction (MI) were also more likely to be enrolled in the DMP-CHD. We found a significant interaction effect for MI and sex, indicating that the association between MI and DMP enrollment is stronger for women than for men.

**Conclusion:**

DMP-enrollees and non-enrollees differ in various factors. Studies analyzing the effectiveness of DMP-CHD should carefully take into account these group differences. Furthermore, the results suggest that the DMP-CHD assessed reaches men better than women.

## Background

In the year 2002, Disease Management Programs (DMPs) were introduced within the German healthcare system, initially for diabetes and breast cancer. The aim was to increase the quality of chronic disease care, based on the provision of coordinated and structured care, following clinical guidelines and by strengthening the patients’ self-care competencies [1].

The first DMPs for coronary heart disease (CHD) were introduced in 2003. By 2014, more than 1.7 million individuals were enrolled in a DMP-CHD [2]. DMPs for CHD aim specifically at: the reduction of mortality; the reduction of cardiovascular morbidity (a reduction of myocardial infarctions (MI) and a reduction in the development of heart failure); and an increase in quality of life [3]. Enrollment in the DMP-CHD is voluntary and without any costs for statutorily health insured. Patients can enroll in the DMP-CHD if they have a documented diagnosis of CHD and are willing to actively participate in the DMP [2].

Due to the enrollment procedures, it can be assumed a) that only certain patients actively decide to enroll in a DMP and/or b) that only certain patients get the recommendation for DMP enrollment from their physician. How strong the effect of self- and/or professional selection is, is still unclear. Comprehensive information on selective enrollment in DMPs is essential for research as well as policy making. First of all, it is important to know if certain population subgroups are not reached although they might benefit from DMPs. Secondly, selective enrollment might influence the results of DMP evaluation [4] and studies trying to evaluate DMPs face the difficulty of selecting suitable control groups [5]. This becomes more important when considering that 14 years after the introduction of DMPs, methodologically solid evidence on their effectiveness is still limited [6]. The mandatory program evaluation according to clause 137f, paragraph 4 in Book Five of Germany’s Social Security Code (SGB V), includes DMP enrollees only, thus a control group is missing and inferences on the effectiveness of DMPs cannot be made.

To overcome the assumed selection bias, many studies analyzing the effectiveness of DMPs apply propensity score matching [5, 7–10]. Yet, those studies often use routinely collected administrative claims data only, and thus, some factors potentially influencing DMP enrollment cannot be included. For instance, it is still rather unclear, how socio-economic factors influence DMP enrollment [11, 12]. On the other hand, analyses relying on survey data only, face the problem of incorrect or incomplete reporting [13].

Most studies on DMPs focused on the DMP for type 2 diabetes (DM2) [4, 6, 9, 14–17]. Little is known about the effects of selective enrollment within the DMP-CHD. To our knowledge, only three publications analyzed selective enrollment in the DMP-CHD. The study by Gapp et al. [18] used data from a MI register, which includes individuals with an acute MI living in the region of Augsburg, southern Germany. Eligible individuals were surveyed and patient characteristics were, if possible, cross-checked with registry data. The study indicated that the odds for DMP enrollment decreased with age and with the time since the last MI. Patients suffering additionally from DM2 were more likely enrolled than those with a cardiac event only [18]. The two publications by Bozorgmehr et al. [11, 12] used data from a cohort study conducted in the German federal state of Saarland. Study participants had either a diagnosis of MI and/or angina pectoris. The focus in both analyses was on inequities in DMP enrollment by education and regional deprivation. In Bozorgmehr et al. [11] the authors reported no horizontal inequities regarding educational attainment, but found that women living in less-deprived areas were significantly more likely to enroll in the DMP-CHD than women in deprived areas. In Bozorgmehr et al. [12] they reported that women generally are less likely to enroll in the DMP-CHD than men but found no effect for education.

The aim of our analyses is to add further knowledge on selective enrollment in the DMP-CHD by identifying factors on patient level that are associated with DMP enrollment. We try to overcome the discussed shortcomings by using a database of survey data linked with individual level claims data by a German sickness fund and by including chronically ill from all federal states of Germany.

## Methods

### Data/sample

Within the project RAC (Responsiveness in ambulatory care [19]), a German-wide postal survey with chronically ill individuals was conducted in October 2013. The sample consisted of persons having CHD who i) were either enrolled in the respective DMP or ii) fulfilled the disease related criteria for enrollment but were not enrolled. The sample was identified based on claims data by a German sickness fund (Techniker Krankenkasse (TK)); from both groups a random sample of 12,999 (not enrolled) and 13,000 (enrolled) individuals was drawn based on defined in- and exclusion criteria (see Table 1). All identified individuals were contacted via post and received the questionnaire, a letter of informed consent regarding the use of administrative claims data and additional informational material (e.g., on data protection issues). Data were only linked and included in the following analyses if both the questionnaire and the signed informed consent were returned. The final dataset comprises i) data from the 2013 postal survey that is linked on individual level to ii) claims data from the TK covering 18 months (from April 2012 to September 2013). Due to the fact that the sample is from one German sickness fund only, it has to be assumed that the results are not representative for the overall German population suffering from CHD. The sample selection, the linkage of survey and claims data as well as the operationalization of variables (including the used ICD-codes) are discussed in more detail in Röttger et al. [19].

**Table 1:** Inclusion and exclusion criteria

Inclusion	Exclusion
For all groups	
• 18 to 90 years at date of initial sample selection (23.9.2013) • Continuously insured at the TK (from 1.1.2012-23.10.2013)	• The insured with increased data security regulation (e.g., former employees of TK) • The insured who are unable to participate in a written survey (e.g., person with legal representative) • Further TK standards (e.g., insured person was contacted for a different survey within last 12 months by TK)
DMP Participants (initial sample *n* = 13,000)	
• Continuously enrolled in indication specific DMP (from 1.1.2012-23.10.2013)	
Non-DMP-Participants (initial sample *n* = 12,999)	
Minimum requirement for identification of coronary heart disease patients based on claims data: • one selected ICD-code (I20-I25, I50) from hospital care within last 36 months OR • one selected ICD-code (I20-I25, I50) from ambulatory care AND one of the selected ATC-codes (C01DA02, C01DA05, C01DA08, C01DA14, C01DA52, C01DX11, C01DX12, C07AA03, C07AA05, C07AA07, C07AB02, C07AB03, C07AB05, C07AB07, C07AB08, C07AB12, C07AB13, C07AG02, C09AA01 - C09AA11, C09BA01 - C09BA09, C09BB05, C09BB10) within last 12 months OR • One selected ATC-code (C01DA02, C01DA05, C01DA08, C01DA14, C01DA52, C01DX11, C01DX12) within last 12 months	• Enrollment in any other DMP (1.1.2012-23.10.2013)

Source: Röttger et al. 2015 [19]

### Analyses

We used binary logistic regression analysis to assess factors associated with DMP enrollment. In the multivariate analysis we specified five models that varied by the included explanatory variables.

### Variable description and rationale for variable selection

DMP enrollment: The status of DMP enrollment was generated from administrative claims data. Enrollment was defined as an individual being continuously enrolled in the DMP-CHD since at least 1.1.2012. Individuals within the group “not enrolled” were not enrolled in the DMP-CHD nor in any other DMP (e.g., for DM2) since at least 1.1.2012.

Socio-demographic and socio-economic factors: The following variables were included in all models: Sex, age, living area (urban vs. rural), net equivalent income, employment status and single household. In addition to these “objective” factors, we also included a variable indicating the subjective socio-economic status (subSES). The variables net equivalent income, employment status, single household and subSES were based on survey data. We calculated the net equivalent income by dividing the monthly net income by the number of household members, weighing the first member with 1 and all other family members with 0.5 [19].^1^

Enrolled in integrated care program: Enrollment in an integrated care program (e.g., in a family physician care model) was assessed from claims data. In contrast to DMPs that primarily focus on the ambulatory care level, integrated care programs mainly aim at cross-sector patient care [20]. We assumed that participants of integrated care programs are more likely to be enrolled in a DMP for two reasons: a) they might be more positive about participating in structured care programs in general; and/or b) may have a GP who is more positive about special care programs in general and is thus more likely to promote DMP enrollment.

Co-Morbidity – generic: As previous studies had discussed that DMP-participants are healthier than non-participants [15], we included various measures of (co-) morbidity: the level of long term care entitlement, the Charlson Score and self-rated health on a scale from 0 (worst imaginable state) to 100 (perfect health). We calculated the ambulatory version of the Charlson Score [21] based on administrative claims data.^2^ All diagnoses, on which variables are based, were identified through ICD-10-Codes from ambulatory and hospital care.

(Co-) Morbidity – disease specific: We included four disease specific morbidity measures: i) a diagnosis of DM2, ii) the level of disease severity, iii) a MI and iv) a congestive heart failure (CHF) diagnosis. DM2 was selected for two reasons: a) it is a frequent comorbidity of CHD and b) a DMP for DM2 exists and thus, persons suffering from CHD and DM2 have a higher chance of being informed about DMPs. The level of disease severity comprised three categories (lowest severity: CHD, other ischemic heart diseases; medium severity: angina pectoris, after MI and without a previous MI; highest severity: acute MI, unstable angina pectoris, other acute ischemic heart diseases). The classification was conducted based on the morbidity-categories and ICD diagnoses used by the German Federal (Social) Insurance Authority [22]. Additionally we included a variable indicating a MI (acute and history of MI) and a variable indicating CHF. Previous studies reported an association between MI and DMP enrollment [11]. Moreover, as the DMP for CHD is offered with an additional module for CHF [2], we assumed that DMP enrollment might be especially interesting for persons suffering from CHF.

A gender bias in the treatment of CHD is frequently discussed, with women and men being treated differently and a greater focus of attention on the treatment of men [23, 24]. We thus included interaction terms between sex and all CHD specific variables (MI, CHF, disease severity).

### Incomplete data, missing values and plausibility checks

Cases with incomplete (only questionnaire or only administrative claims data) or implausible data (e.g., different information on age and sex in questionnaire and administrative data) were excluded from all analyses.

For enrollment in the DMP-CHD a diagnosis of CHD is required. Therefore we only included cases which had according to our data at least one diagnosis related to CHD. Cases without a diagnosis of CHD may have been in the initially selected sample for two reasons: a) for the sample selection a broader time frame was used and b) a rather sensitive approach was used to identify CHD patients (increasing the chance of “false-positive” cases).

We handled missing values in survey data by using multiple imputation. We imputed 15 datasets by fully conditional specification with multinomial logit models and predictive mean matching [25, 26]. All analyses were conducted with the original and the imputed dataset. Except for the descriptive statistics, we only report the results from the imputed datasets. For the logistic regression we report pooled estimates; for all analyses we report a significance level of *p* < 0.05. The multiple imputation was conducted in R (package MICE), the regression analysis in SPSS 22. All study participants gave written informed consent. The study was approved by the responsible ethics committee.

## Results

From 25,999 contacted persons, 8476 persons returned a filled in questionnaire, the signed consent form for data linkage and the linked cases passed the plausibility checks. These cases were included in a (non-) responder analysis, which revealed that DMP-participants, men and older study participants returned questionnaire and consent form more frequently (see Table 2). The response rates across sex and age groups were similar within the groups enrolled in DMP and not enrolled. However, the rise in response rates with age was steeper in the enrolled in DMP group compared to those not enrolled.

**Table 2:** Analysis (non-) responder

	Enrolled in DMP			Not Enrolled			Total		
	initial sample	linked sample	response rate	initial sample	linked sample	response rate	initial sample	linked sample	response rate
Sex									
Female	2,957	952	32.19%	5,002	1,177	23.53%	7,959	2,131	26.77%
Male	10,043	4,189	41.70%	7,997	2,158	26.99%	18,040	6,345	35.17%
Age									
< =60 years	1,963	502	25.57%	4,282	790	18.45%	6,245	1,292	20.69%
61–65 years	1,474	463	31.41%	1,711	440	25.72%	3,185	902	28.32%
66–70 years	1,959	782	39.92%	1,587	434	27.35%	3,546	1,216	34.29%
71–75 years	3,158	1,412	44.71%	2,389	809	33.86%	5,547	2,220	40.02%
76–80 years	2,611	1,234	47.22%	1,669	527	31.58%	4,280	1,763	41.19%
> 80 years	1,835	748	40.76%	1,361	335	24.61%	3,196	1,083	33.89%
Total	13,000	5,141	39.55%	12,999	3,335	25.66%	25,999	8,476	32.60%

Of those 8476 that returned the questionnaire and the consent form, 1407 individuals did not fulfil the official requirement for DMP participation, as they had no relevant diagnosis related to CHD within the six quarters studied. Thus, 7070 cases were included in the following analyses. From those cases that had to be excluded due to not meeting the required diagnosis, 1377 were not enrolled in a DMP and only 30 were enrolled.

In the final sample of 7070 cases, 1092 had one or more missing values that were imputed and subsequently included in all further analysis. Eighty two percent of the imputed sample are male and the mean age is 71.4 (min: 25, max: 90, SD: 9.1) years. The majority of participants lives in a two person household, receives old-age pension, has a net equivalent income between 980€ and 1633€ (i.e., 60–100% of German median) and rates themselves as having a medium socio-economic status (see Table 3).

**Table 3:** Sample characteristics, divided by enrollment-status, reported in % (values after multiple imputation)

Variable	Enrolled in DMP *N* = 5103	Not-Enrolled *N* = 1967	Total *N* = 7070
Socio-demographic and socio-economic factors			
Male	81.6	73.7	79.4
Age in years (mean)	72.37 ± 8.40	68.72 ± 10.35	71.36 ± 9.13
Net equivalent income^a^			
< =979€	13.0 (14.6)	13.5 (15.0)	13.2 (14.7)
980 to 1633€	46.2 (49.6)	38.9 (42.2)	44.2 (47.5)
> 1633 to <2449€	24.0 (25.7)	25.3 (27.1)	24.4 (26.1)
= > 2449€	9.4 (10.1)	14.6 (15.8)	10.8 (11.7)
Missing	7.5	7.7	7.5
Employment/retirement status^a^			
Old-age pension	80.8 (82.7)	66.0 (67.6)	76.6 (78.5)
Employed	11.1 (11.5)	23.6 (24.4)	14.6 (15.1)
Not-employed	5.5 (5.8)	7.4 (8.0)	6.0 (6.4)
Missing	2.7	3.1	2.8
Subjective socio-economic status^a^			
Lowest subSES	19.5 (21.2)	17.8 (19.4)	19.0 (20.7)
Medium subSES	58.8 (63.4)	59.5 (63.6)	59.0 (63.4)
Highest subSES	14.3 (15.4)	15.7 (17.0)	14.7 (15.8)
Missing	2.9	3.0	2.9
Other/None of these subSES	4.5	4.1	4.4
Urban living area	76.6	76.1	76.5
Single household^a^			
1 person in household	15.4 (16.0)	15.3 (15.7)	15.4 (15.9)
> 1 person in household	82.4 (84.0)	82.7 (84.2)	82.5 (84.1)
Missing	2.2	2.0	2.1
Integrated care			
Enrolled in integrated care program	12.5	8.3	11.3
Co-Morbidity - generic			
Level of long term care entitlement			
No entitlement	96.6	97.3	96.8
Level 1	2.7	1.8	2.5
Level 2	0.7	1.0	0.7
Charlson Score			
Charlson Score = 0	10.8	16.8	12.5
Charlson Score = 1	19.9	25.5	21.5
Charlson Score = 2	19.5	18.7	19.3
Charlson Score = 3	16.6	14.6	16.1
Charlson Score > 3	33.1	24.5	30.7
Self-reported health status – Value on VAS (median/mean)	70/64.2 ± 19.0 (*n* = 4,990) (70/64.5)	70/65.4 ± 19.7 (*n* = 1,931) (70/65.7)	70/64.5 ± 19.2 (*n* = 6,921) (70/64.8)
(Co-) Morbidity – disease specific:			
Type 2 Diabetes	26.4	11.6	22.3
Disease Severity			
Lowest severity	54.8	52.4	54.1
Medium severity	26.5	25.5	26.2
Highest severity	18.7	22.2	19.7
Myocardial Infarction (MI)	33.4	27.1	31.7
Congestive heart failure (CHF)	30.7	28.2	30.0

^a^Information from survey data; all other variables derived from administrative claims data; values in brackets = values after multiple imputation; ± standard deviation

Persons enrolled in a DMP have more frequently a reported MI, CHF and a higher number of comorbidities according to the Charlson Score. Considering that all participants suffer from a chronic disease, the self-rated health (mean: 64.5; median: 70) is still rather good (the mean value of the German population was 77.4 in a representative survey in 2002/2003 [27]).

### Multivariate analyses

In the multivariate analyses five binary logistic regression models with varying explanatory variables were specified. Due to the previous imputation of missing values, all models included the full sample of 7070 cases.

Socio-demographic and socio-economic factors: Male sex, higher age and receiving old age pension have higher odds for DMP enrollment in all five models (Table 4). While we found a slight association in Model 1 and 2 between the net equivalent income and DMP enrollment, the association is insignificant when controlling for co-morbidities.

**Table 4:** Results from multivariate analysis - dependent variable: DMP enrollment

Variable	Model 1			Model 2			Model 3			Model 4			Model 5		
	B (SE)	OR	95% CI	B (SE)	OR	95% CI	B (SE)	OR	95% CI	B (SE)	OR	95% CI	B (SE)	OR	95% CI
Constant	**.79 (.17)**	**2.21**		**.76 (.17)**	**2.13**		.83 (.20)	2.29		.64 (.21)	1.89		.61 (.21)	1.84	
Female; *ref.: male*	**-.54 (.07)**	**0.58**	**0.51–0.67**	**-.54 (.07)**	**0.58**	**0.51–0.67**	**-.54 (.07)**	**0.59**	**0.51–0.66**	**-.46 (.07)**	**0.63**	**0.55–0.73**	**-.36 (.10)**	**0.70**	**0.57–0.85**
Age in years; *ref.: <=60*															
61–65	.18 (.12)	1.20	0.95–1.51	.18 (.12)	1.19	0.94–1.51	.19 (.12)	1.21	0.96–1.53	.15 (.12)	1.16	0.90–1.50	.14 (.12)	1.15	0.91–1.47
66–70	**.54 (.15)**	**1.72**	**1.29**–**2.30**	**.54 (.15)**	**1.72**	**1.28-2.30**	**.54 (.15)**	**1.72**	**1.23–2.30**	**.49 (.15)**	**1.62**	**1.20–2.19**	**.49 (.15)**	**1.63**	**1.21–2.20**
71–75	**.48 (.14)**	**1.62**	**1.22–2.15**	**.49 (.14)**	**1.63**	**1.22–2.16**	**.48 (.15)**	**1.62**	**1.22–2.16**	**.46 (.15)**	**1.58**	**1.18–2.11**	**.45 (.15)**	**1.57**	**1.17–2,11**
76–80	**.73 (.15)**	**2.08**	**1.55–2.79**	**.73 (.15)**	**2.08**	**1.55–2.80**	**.72 (.15)**	**2,06**	**1.53–2.80**	**.69 (.16)**	**2,00**	**1.47–2.70**	**.68 (.16)**	**1.97**	**1.45–2.68**
> = 81	**.79 (.16)**	**2.21**	**1.61–3.03**	**.80 (.16)**	**2.22**	**1.62–3.04**	**.78 (.16)**	**2.18**	**1.58–3.00**	**.75 (.17)**	**2.12**	**1.53–2.95**	**.75 (.17)**	**2.12**	**1.53–2.95**
Net equivalent income*; ref.: lowest income < =979€*															
980 to 1633€	.11 (.09)	1.12	0.94–1.33	.11 (.09)	1.12	0.94–1.33	.13 (.09)	1.13	0.94–1.34	.14 (.09)	1.14	0.95–1.36	.13 (.09)	1.14	0.95–1.36
> 1633 to <2449€	-.04 (.10)	0.96	0.79–1.17	-.05 (.10)	0.95	0.79–1.16	-.02 (.10)	0.96	0.80–1.17	-.01 (.10)	0.98	0.80–1.20	-.02 (.10)	0.98	0.80–1.20
= > 2449€	**-.30 (.12)**	**0.74**	**0.58–0.94**	**-.30 (.12)**	**0.74**	**0.59–0.94**	-.20 (.12)	0.76	0.60–0.96	-.17 (.12)	0.82	0.64–1.04	-.21 (.12)	0.81	0.64–1.03
Employment/retirement status; *ref.: old-age pension*															
Employed	**-.41 (.13)**	**0.66**	**0.51–0.85**	**-.41 (.13)**	**0.66**	**0.51–0.86**	**-.43 (.13)**	**0.67**	**0.52–0.87**	**-.44 (.13)**	**0.65**	**0.50–0.85**	**-.43 (.13)**	**0.65**	**0.50–0.85**
Not-employed	-.08 (.14)	0.92	0.70–1.22	-.08 (.14)	0.92	0.70–1.21	-.18 (.15)	0.90	0.67–1.17	-.20 (.14)	0.84	0.63–1.11	-.17 (.15)	0.84	0.63–1.12
Subjective socio-economic status; *ref.: lowest subSES*															
Medium subSES	-.11 (.08)	0.90	0.77–1.04	-.12 (.08)	0.89	0.76–1.03	-.09 (.08)	0.90	0.77–1.05	-.08 (.08)	0.92	0.79–1.07	-.08 (.08)	0.93	0.80–1.08
Highest subSES	-.11 (.10)	0.90	0.73–1.10	-.12 (.10)	0.89	0.73–1.09	-.11 (.11)	0.91	0.74–1.12	-.11 (.11)	0.89	0.73–1.10	-.10 (.11)	0.91	0.74–1.12
Urban living area; *ref.: rural*	-.04 (.07)	0.96	0.84–1.10	-.05 (.07)	0.96	0.84–1.09	-.05 (.07)	0.95	0.83–1.09	-.06 (.07)	0.94	0.82–1.07	-.07 (.07)	0.93	0.81–1.07
Single household; *ref.: >1 person in household*	.03 (.08)	1.03	0.88–1.21	.04 (.08)	1.04	0.88–1.21	.03 (.08)	1.03	0.88–1.21	.03 (.08)	1.03	0.88–1.21	.03 (.08)	1.03	0.88–1.21
Enrolled in integrated care program				**.43 (.10)**	**1.54**	**1.30–1.87**	**.45 (.10)**	**1.54**	**1.27–1.86**	**.45 (.10)**	**1.57**	**1.30–1.91**	**.45 (.10)**	**1.57**	**1.29–1.90**
Level of long term care entitlement; *ref.: no entitlement*															
Level 1							.12 (.21)	1.20	0.80–1.80	.03 (.21)	1.12	0.74–1.69	.31 (.21)	1.03	0.68–1.57
Level 2							**-.73 (.30)**	**0.49**	**0.27–0.89**	**-.83 (.31)**	**0.46**	**0.25–0.85**	**-.83 (.31)**	**0.44**	**0.24–0.81**
Charlson Score; *ref.: Charlson Score = 0*															
Charlson Score = 1							-.10 (.08)	1.18	0.98–1.42	.00 (.80)	1.10	0.91–1.32	.00 (.08)	1.00	0.85–1.17
Charlson Score = 2							**.14 (.08)**	**1.50**	**1.24–1.81**	**.19 (.09)**	**1.33**	**1.10–1.62**	**.17 (.09)**	**1.20**	**1.02–1.42**
Charlson Score = 3							**.18 (.10)**	**1.56**	**1.28–1.92**	**.19 (.09)**	**1.34**	**1.09–1.64**	**.19 (.09)**	**1.20**	**1.01–1.44**
Charlson Score >3							**.15 (.10)**	**1.66**	**1.38–2.00**	**.12 (.10)**	**1.27**	**1.05–1.55**	**.12 (.10)**	**1.12**	**0.92–1.38**
Health related quality of life (VAS 0–100)							−0.00 (.00)	1.00	0.99–1.00	−0.00 (.00)	0.99	0.99–1.00	−0.00 (.00)	0.99	0.99–1.00
DM2; *ref.: no DM2*										**.97 (.08)**	**2.65**	**2.25–3.12**	**.97 (.09)**	**2.64**	**2.24–3.12**
Disease Severity: *ref.: lowest severity*															
Medium severity										**-.43 (.09)**	**0.65**	**0.55–0.77**	**-.28 (.11)**	**0.76**	**0.62–0.93**
Highest severity										**-.72 (.10)**	**0.49**	**0.40–0.59**	**-.59 (.12)**	**0.56**	**0.44–0.70**
MI; *ref.: no MI*										**.76 (.09)**	**2.15**	**1.80–2.56**	**.58 (.11)**	**1.80**	**1.44–2.19**
CHF; *ref.: no CHF*										-.07 (.07)	0.94	0.83–1.07	-.01 (.08)	0.99	0.85–1.14
Interaction terms															
Medium disease severity*female													**-.46 (.19)**	**0.64**	**0.44–0.92**
High disease severity*female													-.46 (.23)	0.63	0.40–1.00
MI*female													**.72 (.23)**	**2.05**	**1.34–3.13**
CHF*female													-.21 (.15)	0.82	0.61–1.10

Bold = *p* < 0.05

Model 1: Model *X* ^*2*^(8) = 311.19–317.80, *p* < .001; Hosmer-Lemeshow *X* ^*2*^(8) = 6.42–16.36, *p* = .04–.60; R^2^ = .065–.066 (Nagelkerke); Model 2: Model *X* ^*2*^(16) = 336.42–343.13, *p* < .001; Hosmer-Lemeshow *X* ^*2*^(8) = 3.10–7.62, *p* = .38–.93; R^2^ = .070–.071 (Nagelkerke); Model 3: Model *X* ^*2*^(25) = 393.96–402.52, *p* < .001; Hosmer-Lemeshow *X* ^*2*^(8) = 4.32–13.7, *p* = .09–.82; R^2^ = .082–.083 (Nagelkerke); Model 4: Model *X* ^*2*^(30) = 611.52–619.52, *p* < .001; Hosmer-Lemeshow *X* ^*2*^(8) = 6.39–10.10, *p* = .26–.60; R^2^ = .124–.126 (Nagelkerke); Model 5: *X* ^*2*^(34) = 623.84–632.55, *p* < .001; Hosmer-Lemeshow *X* ^*2*^(8) = 3.62–12.46, *p* = .13–.89; R^2^ = .127–.129 (Nagelkerke)

Integrated care program: The enrollment in an integrated care program significantly increases the odds of being enrolled in a DMP (OR: 1.54–1.57, *p* < 0.05).

Co-Morbidity – generic: Individuals with higher Charlson Scores, i.e., those with at least two or those with more severe co-morbidities, are more likely to be enrolled. Yet, this effect vanishes for those with even more comorbidities (Charlson Score above 3).

(Co-) Morbidity – disease specific: Individuals with a diagnosed DM2 and individuals with a diagnosed MI have higher odds for DMP enrollment (OR: 2.65, *p* < 0.05; OR: 2.15, *p* < 0.05). Yet, a high disease severity according to the classification of the German Federal (Social) Insurance Authority significantly decreases the odds for DMP enrollment (OR: 0.49–0.65, *p* < 0.05).

**Fig. 1:**
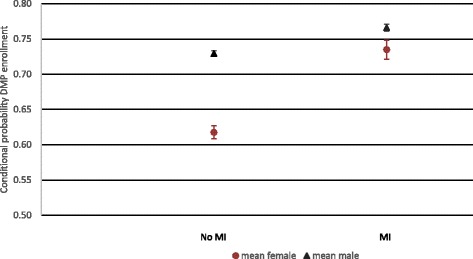
Conditional probability for DMP enrollment by sex and MI

Interaction effects: In addition to the single effects, we find a strong significant interaction effect between MI and sex. While a MI increases the odds of being enrolled in a DMP, the effect is stronger for women than for men. This effect is illustrated in Fig. 1, which depicts the conditional probability for DMP enrollment, by sex and MI.

We conducted the same analyses with the dataset without imputed values using complete case analysis. In these analyses 5978 cases were included and the results were very similar to the results with the imputed dataset.

## Discussion

The aim of this analysis was to identify factors on patient level that are associated with DMP enrollment. Based on administrative claims data of a large German sickness fund, the two groups “DMP-enrollee” and “not enrolled, but fulfills criteria for DMP enrollment” were determined. From these two groups, a random sample each was drawn. Thus, assuming the enrollees and non-enrollees do not differ systematically regarding their characteristics, one could expect to find no differences between the two selected groups. At the same time, it can be assumed that differences between the groups indicate that these are either due to “selective enrollment” or that the DMP participation/non-participation led to these differences.

We found significant effects for age and sex, with higher age and a male sex increasing the odds for DMP enrollment. Our findings regarding sex are consistent with the results reported by Bozorgmehr et al. [12]. However, both findings are different to the study results by Gapp et al. [18]. The differences in the effect of age are probably due to the year of the survey/study (2006 vs. 2013). While the DMP-CHD was still rather new during the survey of Gapp et al., individuals suffering from CHD had have up to 9 years to enroll in the DMP at the point of our study. Differences considering the gender distribution (Gapp et al. did not find a significant association between sex and enrollment) are probably resulting from distinct CHD morbidities [18]. While the study by Gapp et al. [18] included exclusively individuals with a history of a MI, we find that differences between men and women are especially strong for those who did not have a diagnosed MI.

As assumed, the association between disease severity and DMP enrollment is affected by the patient’s sex: for women the association between MI and DMP enrollment is a lot stronger than the same association for men. Thus, it seems that women need a worse disease state then men, before they consider or are asked to enroll in the DMP-CHD. These results support existing studies focusing on gender differences in health care settings and particularly in the treatment of CHD [23, 28].

Further morbidity indicators are significantly associated with DMP enrollment: a higher level of disease severity decreased the odds for DMP enrollment whereas a diagnosed MI increased the odds for enrollment. Thus, the DMP-CHD seems to be especially interesting for patients with a low severity of CHD and for patients who suffered from a MI. In contrast, suffering from unstable angina pectoris (disease severity high) reduces the odds for DMP enrollment. This effect might be explained by the initial aims of the DMP, which is to help patients managing their disease and to avoid a worsening of the disease. Thus, those who are still in a lower disease severity might have a better chance to profit from the DMP enrollment than those with a high severity (e.g., patients that already suffer from unstable angina pectoris). In addition, the required “active participation” of the patients for DMP enrollment, might be a barrier for very ill patients.

The results indicate a strong association for DM2 and DMP-CHD enrollment. As previously discussed, this is probably due to the fact that a DMP program for DM2 exists, and thus, the chances to get to know about DMPs at all are higher for individuals having both conditions.

Employment status and DMP enrollment also correlate, with employed individuals being less likely to enroll in a DMP. A possible reason is that employed individuals cannot spend or do not want to spend as much time with their disease management as, for instance, individuals receiving old-age pension. This explanation is supported by studies showing that employed individuals also report to forgo care more often than others [29].

In the interpretation of our results several limitations have to be considered. First of all, although the study includes patients from all over Germany, all of them are insured at one sickness fund (TK). Hence, our sample is not representative of all CHD patients in Germany, and the generalizability of our results may be limited [30]. In addition, our results might be affected by differences in the survey-response behavior. It is possible that certain groups of patients did not participate in our study e.g., very ill individuals. However, we were able to analyze the (non-) response rates for age, sex and DMP enrollment. These revealed an overall higher response rate for individuals enrolled in a DMP, but the comparison of the response rates in relation to sex and age split by DMP enrollment status, showed rather similar results (e.g., women had in both groups a lower response rate than men).

We used cross-sectional survey data from October 2013 and administrative claims data from 01 April 2012 to 30 September 2013, while the date of DMP enrollment was any time before 01 January 2012. Thus, for some variables we do not know if they influence the DMP enrollment or if enrollment influences the occurrence of e.g., a certain diseases. Yet, it is rather unlikely that e.g., DMP enrollment leads to more MI in women. The interpretation that women are more frequently enrolled if they had a history of MI seems therefore legitimate. As in all analyses using administrative claims data, it has to be considered that the primary reason for data collection was different to the aim of the conducted analyses. Especially in the comparison of DMP-enrollees and non-enrollees it has to be considered that the medical documentation for patients within a DMP might be slightly better.

## Conclusion

This is the first study that comprehensively analyses the association between patient characteristics and DMP-CHD enrollment for patients from all over Germany. Although this study focuses solely on selective enrollment within German DMPs, selective enrollment may also exist in other voluntary care programs (e.g., in the context of integrated care). As care programs address a predefined target population, some intended selection effects are desirable. The problem of adverse selective enrollment arises when the selection effects are not the defined target effects but disadvantage certain population groups.

The results indicate that DMP-enrollees and non-enrollees differ in various factors, e.g., sex, age, employment-status, (co-) morbidities and that selective enrollment may exist. Studies evaluating the DMP-CHD should carefully take into account the differences between both groups. The results indicate that the CHD assessed reaches men better than women. These findings needs further evaluation, as they suggest that the program either suits men better than women or that it is promoted to men more successfully than to women.

## Abbreviations

CHD: Coronary heart disease
DM2: Type 2 diabetes
DMP: Disease management program
MI: Myocardial infarction
subSES: Subjective socio-economic status
TK: Techniker Krankenkasse
CHF: Congestive heart failure

## Footnotes

1 We did not differentiate between adults and children under 14 years of age (as it is done in the modified OECD scale [31]), as the survey only included a question regarding the number of children under 18 years of age. But due to the high mean age of the sample, we assume a very small number of under 14 year old children in our sample.

2 We excluded the diseases MI, CHF and DM2 from the calculation of the Charlson Score.
